# Mechanical and Physical Properties of Oriented Strand Lumber (OSL): The Effect of Fortification Level of Nanowollastonite on UF Resin

**DOI:** 10.3390/polym11111884

**Published:** 2019-11-14

**Authors:** Vahid Hassani, Hamid R. Taghiyari, Olaf Schmidt, Sadegh Maleki, Antonios N. Papadopoulos

**Affiliations:** 1 Wood Science and Technology Department, Faculty of Materials Engineering and New Technologies, Shahid Rajaee Teacher Training University, Tehran 14115, Iran; hassanivahid1988@gmail.com; 2 Department of Wood Biology, University of Hamburg, Leuschnerstr. 91, 21031 Hamburg, Germany; olaf.Schmidt@uni-hamburg.de; 3 Department of Wood Science and Technology, Faculty of Natural Resources, Tarbiat Modares University, Tehran 14115, Iran; s.maleki33@gmail.com; 4 Department of Forestry and Natural Environment, International Hellenic University, Laboratory of Wood Chemistry and Technology, GR-661 00 Drama, Greece

**Keywords:** oriented strand lumber (OSL), nanowollastonite, mechanical and physical properties, UF resin

## Abstract

The aim of this work is to investigate the effect of the fortification level of nanowollastonite on urea-formaldehyde resin (UF) and its effect on mechanical and physical properties of oriented strand lumbers (OSL). Two resin contents are applied, namely, 8% and 10%. Nanowollastonite is mixed with the resin at two levels (10% and 20%). It is found that the fortification of UF resin with 10% nanowollastonite can be considered as an optimum level. When nanowollastonite content is higher (that is, 20%), higher volume of UF resin is left over from the process of sticking the strips together, and therefore is absorbed by wollastonite nanofibers. The mechanism involved in the fortification of UF resin with nanowollastonite, which results in an improvement of thickness swelling values, can be attributed to the following two main factors: (i) nanowollastonite compounds making active bonds with the cellulose hydroxyl groups, putting them out of reach for bonding with the water molecules and (ii) high thermal conductivity coefficient of wollastonite improving the transfer of heat to different layers of the OSL mat, facilitating better and more complete resin curing. Since nanowollastonite contributes to making bonds between the wood strips, which consequently improves physical and mechanical properties, its use can be safely recommended in the OSL production process to improve the physical and mechanical properties of the panel.

## 1. Introduction

Oriented strand lumber (OSL) is a structural panel with consistent properties from one unit to another, which is capable of handling large loads. OSL is made by aligning long strands of wood in parallel and binding them together using adhesives, pressure, and heat. It replaces softwood timber in some residential building applications, but because it can attain dimensions not possible for a single piece of wood, it has additional applications in nonresidential construction. OSL is also used for industrial purposes such as furniture manufacturing [1].

A major difference in the performance of structural panels compared to solid wood is the greater thickness swell that occurs when the panels are exposed to relative humidity and/or direct contact with water. This is due to the higher pressure required to consolidate the panel mat. The issue of improving the thickness swell of panels like particleboard, fiberboard, and oriented strand board (OSB) has been a topic of interest for many researchers [2,3,4]. Briefly, there are several treatment methods that can be divided into three different means of application: pretreatment, post-treatment, and production technology. The first group includes methods that involve treatments applied to furnish before panel hot pressing, such as particle presteaming and chemical or thermal modification of particles [2,3,4,5,6,7]. The second group comprises methods applied in the consolidated panels, and thermal treatment is the most common [8,9]. Last, production technology methods involve those related to resin content improvement, mat-forming type, platen and press temperature, and water repellent application [10,11,12,13,14,15,16,17]. These days, the application of water repellents is a common practice. However, a major challenge in the manufacture of water-resistant, wood-based panels is identifying compatible combinations of water-repellent chemicals and adhesives. Waxes are commercially used to improve the water repellency of wood-based panels [18]. Oils, such as silixane systems and acrylic elastomeric coating systems, have been used to improve the weather-proofing of siding panels [19,20]. The use of silanes, silicones, and siloxanes is well established for glass fiber-reinforced plastic composites, but their use in wood-based panels is rather limited mainly due to their water insolubility and tendency to form silica deposits [21].

An attractive science, nanotechnology seems to have remarkable potential to create products of a new generation with enhanced properties [21,22]. The change in material properties is primarily due to the large interfacial area that is developed per unit of volume, since the level of added particles is reduced to nanometers. Nanomaterials enhance the properties of the original material, show great compatibility with traditional materials, and cause limited alteration of their original features [23,24]. Their use in wood has the objective of improving its physical and mechanical properties and its durability against microorganisms, since it is generally acceptable that nanosized metals and minerals interact with bacterial elements, gradually leading to cell death [23,24,25,26] or even to the disruption of enzyme function [25,26,27]. An excellent review of the application of nanotechnology on wood science was recently performed [21,28].

The approach of this work is to look at ways of improving the dimensional stability of OSL through nanotechnology. In particular, the aim of this study is to investigate the effect of the fortification level of nanowollastonite on urea-formaldehyde resin (UF), and its effect on mechanical and physical properties of oriented strand lumbers.

Wollastonite (a silicate mineral, CaSiO_3_) makes bonds with cell-wall polymers of wood [29,30]. The adsorption energy of nanowollastonite (NW) on cellulose surface was reported to be as high as −6.6 eV, formed between Ca in NW and hydroxyl groups in cellulose chains [29]. In another study, the optimal adsorption distance of 2.5 Å in OH_cellulose_ Ca was reported as the most important factor in the formation of the strong bond [31]. The formation of these bonds resulted in significant improvement in physical and mechanical properties in composite panels treated with NW suspension [32]. Moreover, the high thermal conductivity coefficient of wollastonite, along with its noncombustible mineral nature, was reported to ultimately cause an improvement in fire properties [30]. In addition, NW acted as a reinforcement agent in polyvinyl resin, significantly improving shear bond strength [33]. However, the application of NW in OSL panels has not yet been tested. Therefore, in the present research project nanowollastonite is used at two consumption levels to find out its effects on the physical and mechanical properties of OSL produced with two resin contents (8% and 10%). 

## 2. Materials and Methods

### 2.1. Panel Production

Poplar strips were prepared from 15-year-old poplar trees (*Populus nigra*) cut from Khoy city, located in Azarbayjan Sharghi Province, Iran. The mean density of the poplar logs was 0.42 g/cm^3^. The logs were first peeled and dried to a final moisture content of 6%. They were then stripped using stripper equipment produced by Iran-Randeh Co. (Tehran, Iran). Dimensions of the strips were 150 mm × 20 mm × 1 mm. The length of the strips was parallel to the longitudinal direction of the logs. Strips were kept in an oven for 48 h at 50 °C and put in sealed plastic bags to prevent moisture absorption from the air. UF, with 200–400 cP in viscosity, 47 s of gel time, and 1.277 g/cm^3^ in density and containing 62% solids, was purchased from Iran Choob Co. (Ghazvin, Iran). As a hardener, 2% ammonium chloride was mixed with the resin before applying it to the strips. Ammonium chloride is a common and effective hardener used for accelerating UF resin curing. Ammonium chloride was chosen for consistency with the industry sector, as most wood-composite manufacturing factories in Iran use ammonium chloride as a hardener, although its use is regulated in some countries due to potential health hazards. The main effect of ammonium chloride on UF resin curing is that it catalyzes the reactants in UF-resin systems. The pH value and the gel time of UF resins decrease with increasing catalyst and resin solid contents and decreasing pH [34,35]. The resin was sprayed on the strips in a rotary drum, and the strips were then manually set in the forming. OSL panels were produced with two resin contents, namely, 8% and 10%. Mats were hot pressed for 10 min. The temperature of the hot-press platens was 170 °C. The pressure of the hot press was 50 kg/cm^2^. Density of all OSL panels was 0.8 g/cm^3^. Dimensions of panels were 45 cm × 45 cm with 16 mm in thickness. For each treatment, five replicates were produced. After manufacture, the boards were conditioned at 25 °C and 45% relative humidity. Values for mechanical properties, namely, internal bond (IB), modulus of rupture (MOR), modulus of elasticity (MOE), tension parallel to grain, shear strength parallel to grain, and impact bending were then determined according to procedures defined in the American standard for particleboards (ANSI A208.1-1998) [36]. ASTM standards were selected for the determination of mechanical properties because they are more stringent than the EN standards, in terms of mechanical properties. However, values for physical properties, namely, thickness swelling (TS) and water absorption (WA), were determined according to procedures defined in the European Union standards (EN 317-1993) [37] since they are less stringent than the ANSI standards, bearing in mind that UF resin was used as a binder. In any case, it is not the scope of this study to look at the data in light of industry standards.

### 2.2. Nanowollastonite Application 

**Figure 1:**
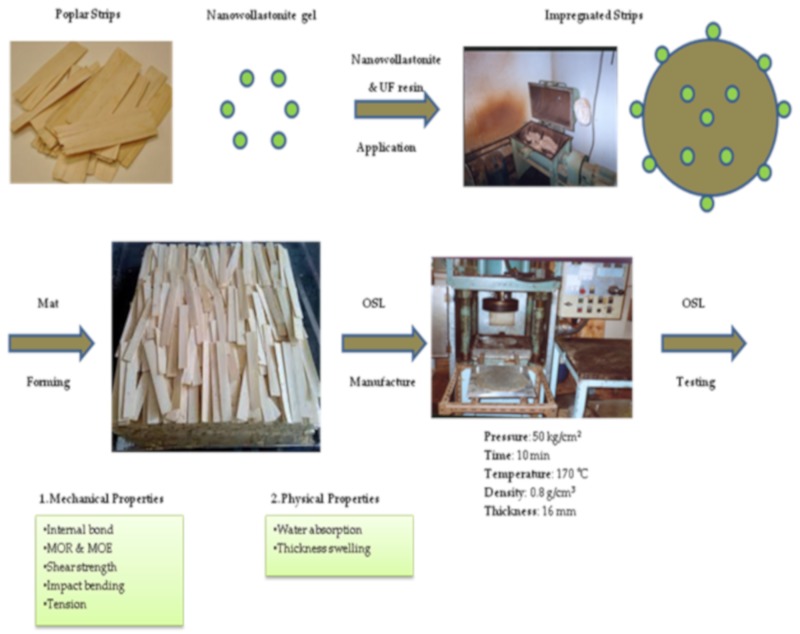
Flow diagram of the experimental procedure.

Nanowollastonite gel was produced in cooperation with Mehrabadi Mfg. Co. in Tehran, Iran. The size range of wollastonite nanofibers was measured as 30–110 nm. Specifications of wollastonite combination are indicated in Table 1. NW was mixed with the urea formaldehyde resin for 30 min by a magnetic stirrer for each load of the drum mixer. The mixture was then sprayed onto the wood fibers in a drum mixer 50 cm in diameter. Consumption level of wollastonite gel was 10% and 20% based on the dry weight of wood strips. The flow diagram of the experimental procedure is depicted in Figure 1.

### 2.3. Thermal Conductivity Measurement 

**Figure 2:**
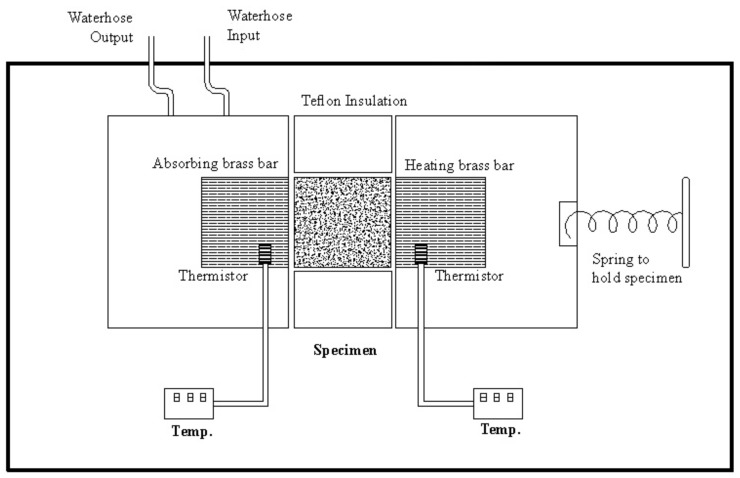
Schematic drawing of the apparatus for measurement of thermal conductivity [38].

**Figure 3:**
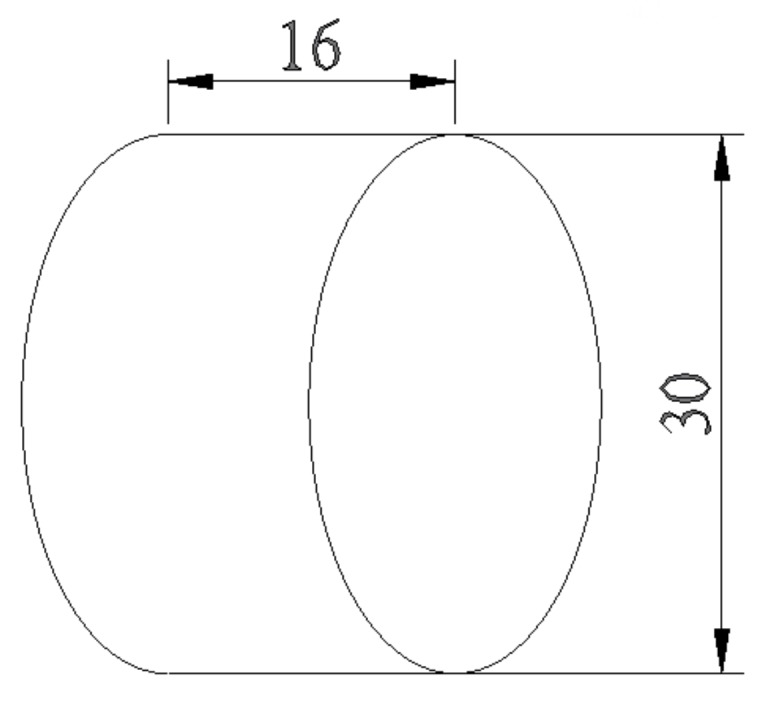
Schematic drawing of cylindrical specimens positioned in between the heating brass bar and absorbing brass bar (dimensions in mm) [38].

Thermal conductivity coefficient of OSL specimens was calculated based on Fourier’s law for heat conduction, using an apparatus by Iranian Precise System Co. (IPS. Tehran, Iran) (Figure 2). Circular specimens were cut 30 mm in diameter and 16 mm in length (Figure 3), and all parts of the specimens were covered with silicone adhesive for better insulation. Specimens were positioned in a Teflon holder to be placed between the heating and absorbing brass rods. The heating brass bar heated the specimen from one side at 130 °C (Figure 2), while the other face of the cylindrical specimen was touched by the absorbing brass bar. The heating continued until the thermistor read a constant temperature. In order to measure the rate of heat transfer, temperature at the middle of the specimen was read and registered at 5 s intervals. Thermal conductivity was then calculated using Equations (1) and (2). Temperatures were measured with 0.1 °C precision.

(1) $$Q=KA\frac{T_{1}-T_{2}}{\Delta_{x}}$$

(2) $$K=\frac{Q\times L}{A\times \Delta T}$$

where

k = Coefficient of thermal conductivity (W.m^−1^.K^−1^)
Q = Heat transfer (W)
L = Specimen thickness (m)
A = Cross section area of specimens (m^2^)
ΔT = Temperature difference (T1–T2) (°k)

### 2.4. SEM Imaging

SEM imaging was carried out at the thin-film laboratory, FE-SEM lab (Field Emission), School of Electrical and Computer Engineering, University of Tehran. A field-emission cathode in the electron gun of a scanning electron microscope provided narrower probing beams at low as well as high electron energy, which improved the spatial resolution and minimized charging and damage to the specimens. As wood is a nonconductive material, a gold sputtering thickness of 6–8 nm was applied on the surface of the specimens prior to SEM imaging.

### 2.5. Statistical Analysis

SAS software program (Statistical Analysis System, version 9.2, 2010) was used to conduct a two-way analysis of variance (ANOVA) to discern significant differences among treatments at the 95% level of confidence. Grouping of similar treatments was made by Duncan’s multiple range test. Hierarchical cluster analysis with Ward’s method was performed using SPSS/20 to clarify similarities and dissimilarities among different treatments based on more than one property at the same time. In this analysis, the scaled indicator determines the degree to which the analyzed treatments are similar or different. Fitted-line and scatter plots were made using Minitab software, version 16.2.2.

## 3. Results and Discussion

### 3.1. Physical Properties

**Figure 4:**
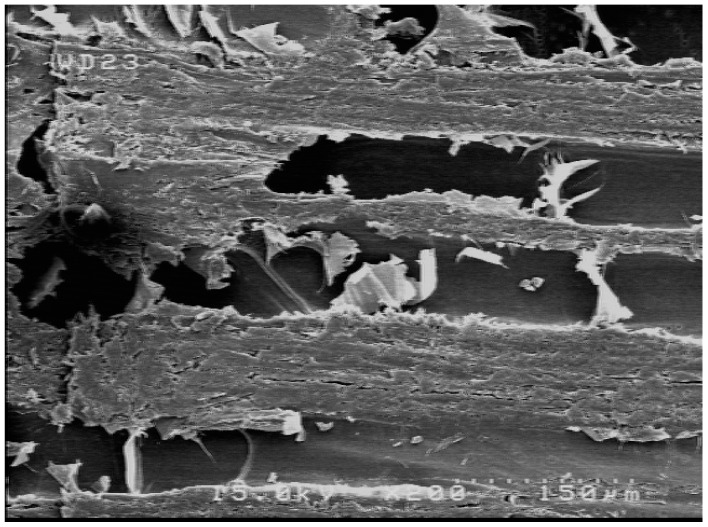
SEM image of the surface of wood strips to be glued and stuck together.

**Figure 5:**
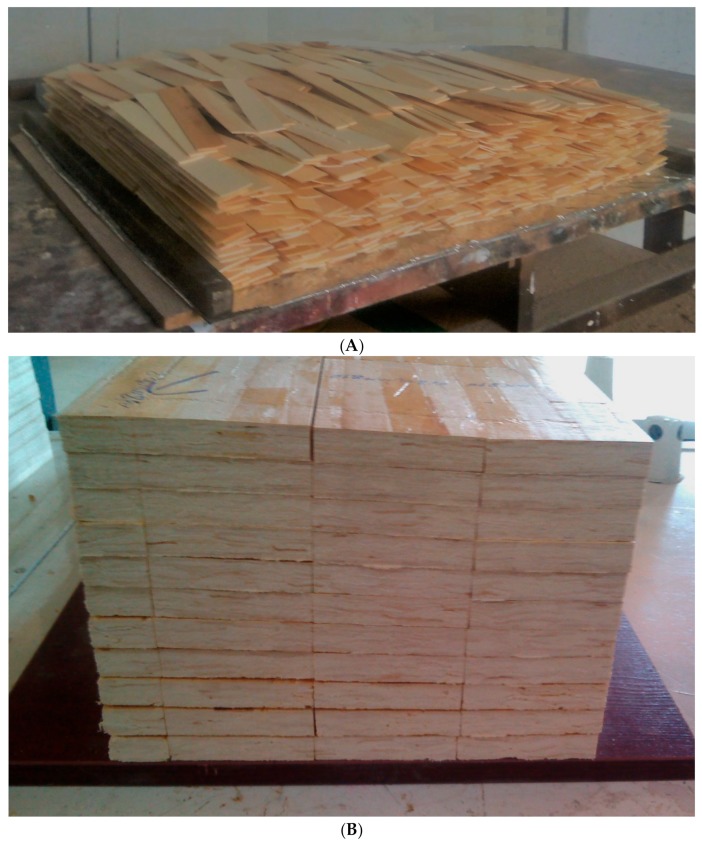
Photos of a wood-strip mat before being hot pressed (**A**), and cut-to-size and ready specimens for testing (**B**).

**Figure 6:**
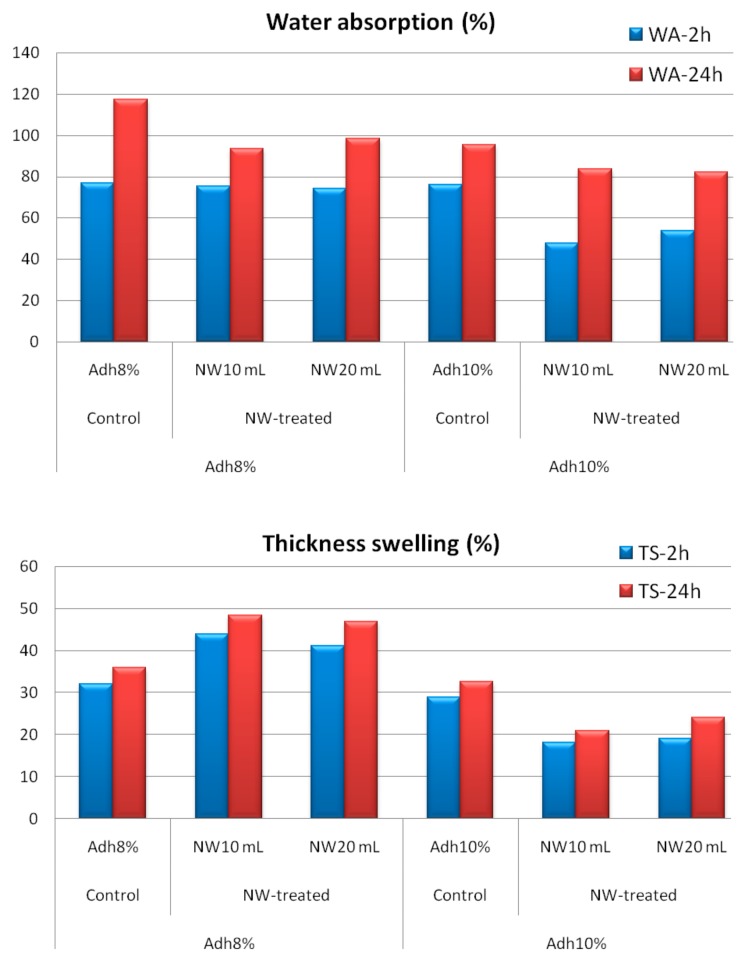
Water absorption and thickness swelling values of control and NW-treated OSL panels as affected by resin and nanowollastonite content (Adh = resin content; NW = nanowollastonite content).

Figure 4 shows an SEM image of the surface of wood strips. Figure 5 demonstrates a wood-strip mat before being hot pressed (A), as well as specimens cut to size and ready for tests (B). Figure 6 depicts the effect of resin content on water absorption and thickness swelling of OSL. As expected, higher resin content resulted in improved properties; however, this improvement was not significant at the 0.05 probability level. Figure 6 also reveals the effect of fortification level of nanowollastonite on UF resin, in both properties. The fortification of UF resin with wollastonite gel (at 10% and 20% based on the dry weight of wood strips) did not significantly affect the water absorption, and this was true for both resin contents applied in this study, namely, 8% and 10%. This was also the case for the thickness swelling in boards manufactured with 8% resin content. However, in boards made with 10% resin content, the situation was different. The fortification of nanowollastonite on UF resin resulted in significant improvement in thickness swelling values, although the values of boards made with 20% nanowollastonite were slightly better than the ones made with 10% nanowollastonite. This requires explanation. The mechanism involved in the fortification of UF resin with nanowollastonite, which resulted in an improvement in thickness swelling values, can be attributed to the following two factors: (i) individual strips in the OSL matrix were better connected to each other through a network of bonds formed between the nanowollastonite compounds and wood-strips functional groups, mainly cellulose hydroxyl groups, putting them out of reach for bonding with the water molecules [31,32,33], and (ii) high thermal conductivity coefficient of wollastonite improved the transfer of heat to different layers of OSL mat [39], facilitating better and more complete resin curing. In a recent study, nanowollastonite was applied at 2, 4, 6, and 8%, based on the dry weight of wood fibers, and its effect on thermal conductivity of the medium density fiberboards was reported. It was found that nanowollastonite significantly increased the thermal conductivity of the panels, which in turn resulted in improved thickness swelling values of the MDF (Medium Density Fiberboards) boards. In particular, the use of 8% nanowollastonite contributed to better heat transfer in a way that thermal conductivity coefficient was increased by more than 29% [39].

### 3.2. Mechanical Properties

**Figure 7:**
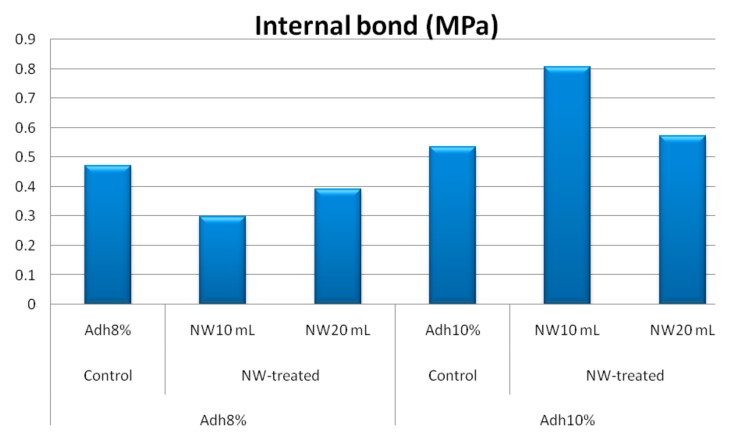
Internal bond strength values of control and NW-treated OSL panels as affected by resin and nanowollastonite content.

Figure 7 depicts the effect of resin content on internal bond of OSL. As expected, higher resin content levels resulted in improved property; however, this improvement was not significant at the 0.05 probability level. Figure 7 also reveals the effect of fortification level of nanowollastonite on UF resin.

The fortification of nanowollastonite in boards made from 8% UF resin resulted in significant reduction in bond strength. At this resin content, it seems that nanowollastonite caused poor wetting of wood strips since the majority of failures were due to the resin and not to the wood. Another possible explanation for this behavior may be the absorption or gathering of resin molecules by wollastonite nanofibers, preventing them from being active in the process of sticking the strips together [39,40,41].

However, at higher resin content (10%), the internal bond strength was increased. This increase was statistically significant at the 0.05 probability level, when 10% nanowollastonite was applied. Therefore, it appears that the increased resin content caused significant wetting of wood strips. Furthermore, an increase in the thermal conductivity coefficient by nanowollastonite [39] caused better curing of the resin in the core section of the mat, resulting in a higher internal bond. This observation demonstrated that 10% of nanowollastonite can be considered an optimum level. In fact, when nanowollastonite content was higher (that is, 20%), higher volume of UF resin left over from the process of sticking the strips together, and therefore was absorbed by wollastonite nanofibers. However, 10% of NW seems to be high enough to improve the properties and low enough not to interfere with the resin.

**Figure 8:**
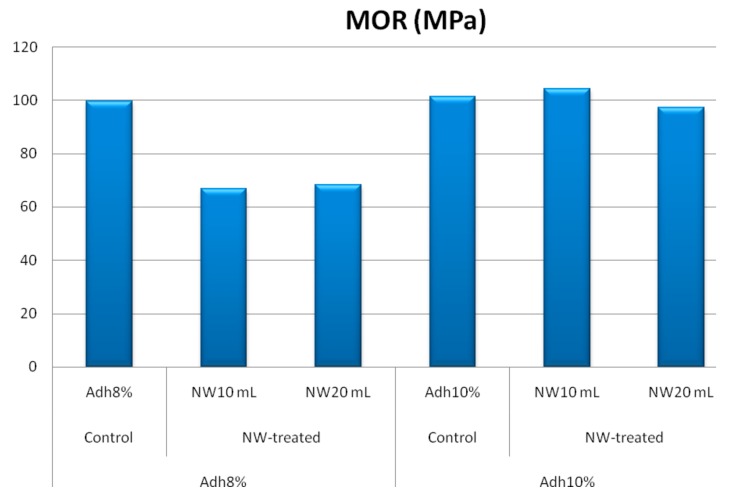
MOR (modulus of rupture) values of control and NW-treated OSL panels as affected by resin and nanowollastonite content.

**Figure 9:**
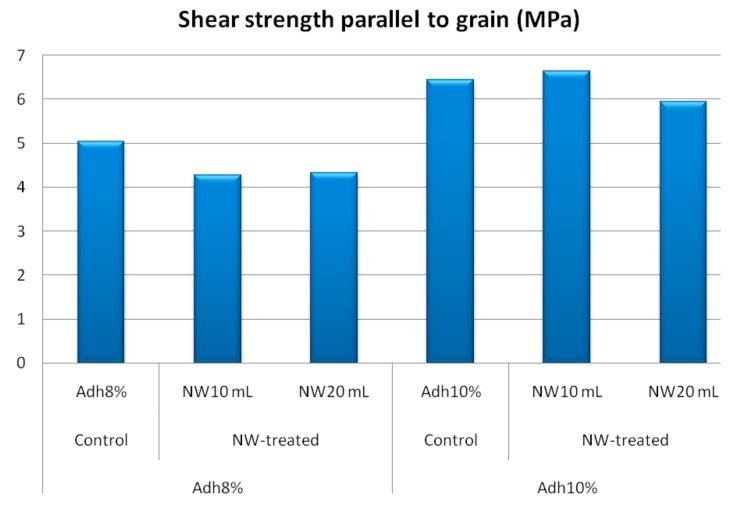
Shear strength values of control and NW-treated OSL panels as affected by resin and nanowollastonite content.

**Figure 10:**
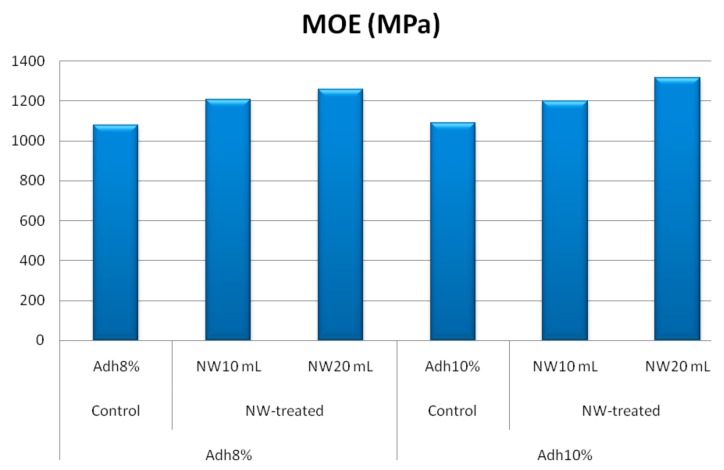
MOE (modulus of elasticity) values of control and NW-treated OSL panels as affected by resin and nanowollastonite content.

**Figure 11:**
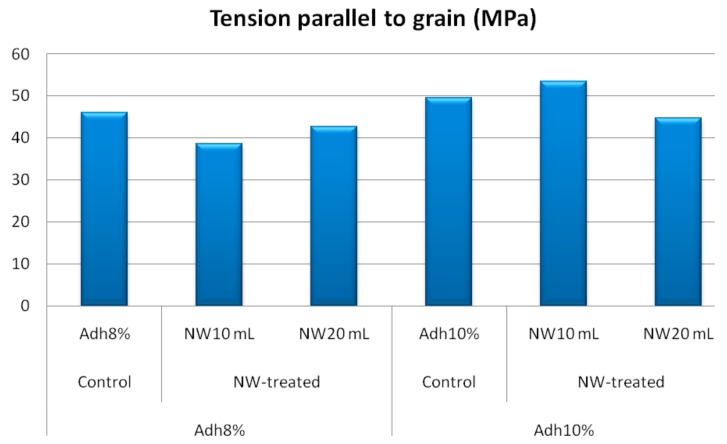
Tension strength values of control and NW-treated OSL panels as affected by resin and nanowollastonite content.

**Figure 12:**
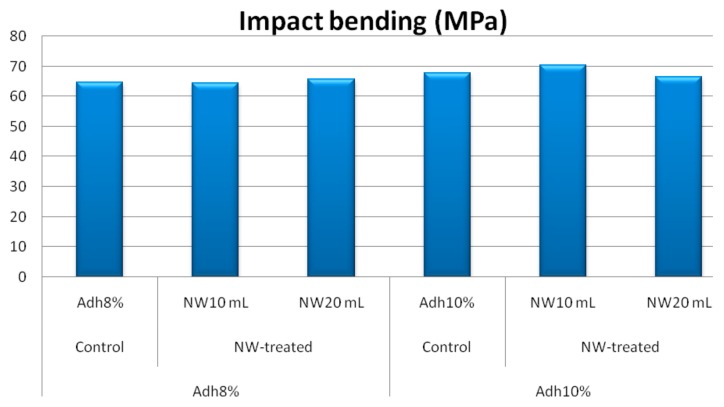
Impact bending values of control and NW-treated OSL panels as affected by resin and nanowollastonite content.

Modulus of rupture and shear strength parallel to grain at low-resin content showed similar behavior to the internal bond strength, as depicted in Figure 8 and Figure 9, respectively. At high-resin content, the fortification with nanowollastonite did not significantly affect these two properties. The other mechanical properties, namely, modulus of elasticity, tension parallel to grain, and impact bending, were not significantly affected by either resin content or the fortification of nanowollastonite, as illustrated in Figure 10, Figure 11 and Figure 12, respectively.

**Figure 13:**
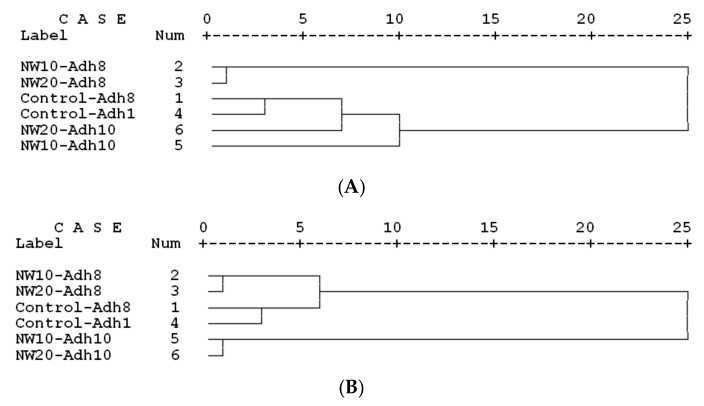
Cluster analysis of the six OSL panels produced based on the mechanical (**A**) and physical (**B**) properties.

Cluster analysis, based on the mechanical properties, clearly showed distinctly different clustering of nanowollastonite-treated panels with 8% resin content (NW10-Adh8 and NW20-Adh8 treatments) (Figure 13A). Both control treatments with resin contents of 8% and 10% were closely clustered together. NW-treated panels with 10% resin contents were somehow clustered similarly and close to the control treatments. This clustering pattern indicated that the addition of NW at lower resin content of 8% had significantly negative effects on the overall mechanical properties, and thus is not recommended, while at the higher resin content of 10%, the addition of NW had some significantly positive effects on individual properties.

On the other hand, cluster analysis based on the physical properties revealed significantly different clustering of NW-treated panels with 10% resin content (Figure 13B). Both control treatments were closely clustered to NW-treated panels with 8% resin content. This indicated that the overall physical properties of NW-treated panels with 8% resin content were similar to the control panels, although the addition of NW had negative effects on the thickness swelling. Moreover, NW can significantly improve physical properties of OSL panels when resin content is high enough (10%).

### 3.3. Thermal Conductivity Coefficients

**Figure 14:**
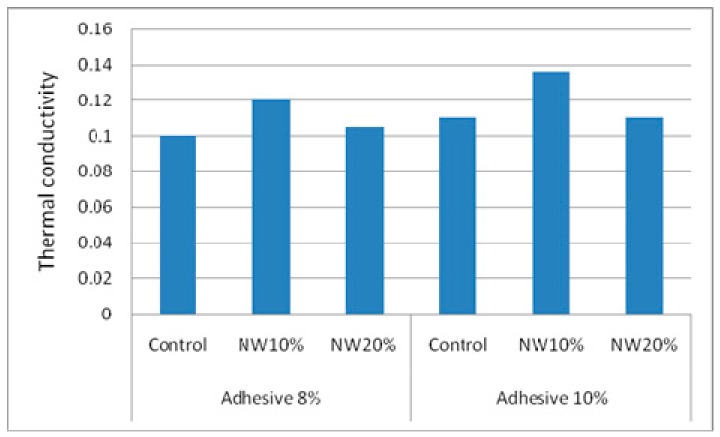
Thermal conductivity coefficients of control and NW-treated OSL panels as affected by resin and nanowollastonite content.

Figure 14 depicts the effect of the addition of nanowollastonite on thermal conductivity coefficient of different specimens. The highest and lowest thermal coefficients were found in NW10%-Adh10% and Control-Adh8% treatments, respectively. The increase in adhesive content from 8% to 10% resulted in an increase in thermal conductivity in all treatments, although the increase was not statistically significant in any of them. The increase was attributed to the better integrity of wood strips when stuck together, as well as the higher connecting points among them.

The addition of nanowollastonite at both NW contents (10% and 20%) made thermal conductivity increase. However, the increase was statistically significant only in treatments with 10% of NW. Treatments with 20% of NW did not demonstrate significant increase in comparison to their control counterparts. Here, NW particles seemed to absorb much resin, negatively interfering with the main role of the resin, which was to stick the strips together.

Based on the above discussion, it seems that the fortification of UF resin with 10% nanowollastonite can be considered an optimum level. When nanowollastonite content was higher (that is, 20%), a higher volume of UF resin left over from the process of sticking the strips together was absorbed by wollastonite nanofibers. However, a NW content of 10% seems to be high enough to improve the panel properties and low enough not to interfere with the resin. Therefore, in future research projects, nanowollastonite contents with intervals close to 10% are recommended to obtain a better scope of the best NW content option.

## 4. Conclusions

The aim of this work was to investigate the effect of fortification level of nanowollastonite on UF resin and its effect on mechanical and physical properties of oriented strand lumbers. Two resin contents were applied, namely, 8% and 10%. Nanowollastonite was mixed with the resin at two levels of 10% and 20%. It was found that the fortification of UF resin with 10% nanowollastonite can be considered as an optimum level. When nanowollastonite content was higher, a higher volume of UF resin left over from the process of sticking the strips together was absorbed by wollastonite nanofibers. Since nanowollastonite contributes to making bonds between the wood strips, which consequently improves their physical and mechanical properties, its use can be safely recommended in the OSL production process to improve the physical and mechanical properties of the panel.

## Figures and Tables

**Table 1:** Composition of the nanowollastonite gel used [22,23,25].

Nanowollastonite Compounds	Content by Mass (%)
CaO	39.77
SiO_2_	46.96
Al_2_O_3_	3.95
Fe_2_O_3_	2.79
TiO_2_	0.22
K_2_O	0.04
MgO	1.39
Na_2_O	0.16
SO_3_	0.05
Water	The rest
